# A Qualitative Analysis on the Effectiveness of Peer Feedback in Team-Based Learning

**DOI:** 10.1007/s40670-023-01813-z

**Published:** 2023-06-20

**Authors:** Sarah Lerchenfeldt, Suzan Kamel-ElSayed, Gustavo Patino, Stephen Loftus, David M. Thomas

**Affiliations:** 1 Department of Foundational Medical Studies, Oakland University William Beaumont School of Medicine, Rochester, MI USA; 2 Department of Medical Education, Western Michigan University Homer Stryker MD School of Medicine, Kalamazoo, USA

**Keywords:** Peer feedback, Peer assessment, Team-based learning (TBL), Medical education

## Abstract

**Introduction:**

There is limited information on medical students’ perceptions of peer feedback in team-based learning (TBL), both in terms of its value and how it has affected them as they move forward in their careers. The primary goals of this study were to examine students’ perceptions about their peer feedback experiences throughout medical school and into residency and to identify areas for improvement to develop a more valuable experience.

**Materials and Methods:**

This study utilized exploratory qualitative research. A total of six focus group sessions were conducted, in which each group consisted of medical students or residents. All participants were asked for their thoughts about peer feedback using semi-structured interviews. The sessions were transcribed and thematic analysis of student responses was completed by independent reviewers.

**Results:**

A total of 11 first-year, 12 second-year, 12 rising third-year, and 10 rising fourth-year medical students participated in the focus groups. In addition, three graduates participated in the study. Overall, four key themes were identified regarding the peer feedback experience. These included (1) preparation and training, (2) procedure and implementation, (3) evaluation of student feedback, and (4) student considerations.

**Discussion:**

The participants indicated that the idea of providing and receiving effective peer feedback throughout the medical school curriculum was a valuable experience. This analysis raised awareness about several potential areas of difficulty for students in regard to the peer feedback process used in TBL. Quality improvement initiatives may include educating students about the use of constructive feedback, adding self-reflection, or using oral instead of written feedback.

## Introduction

In this project, we conducted an in-depth exploration of student perceptions about giving and receiving peer feedback in the team-based learning (TBL) setting. According to the theory of constructivism, learning is a process that includes active participation, in which students construct their own understanding of the material through reflection on past and current experiences and actions to effectively use information and improve their knowledge and skills [1, 2]. While rooted in cognitive constructivism, a more recent view known as the social constructivist theory explains that learning and understanding are social and dependent on interactions with others, including peers [3].

TBL, a commonly used instructional strategy in health professions education, is grounded in constructivist learning theory, in which students work collaboratively with teams through real-life clinical cases [1]. Peer evaluation is one of the essential elements of TBL, where students are expected to provide feedback to their teammates about their contributions to the team’s success [4]. According to Cestone and colleagues, peer evaluation is necessary as it provides information to help individual students improve team performance, as well as develop interpersonal and team skills [5]. By giving and receiving peer feedback, students are encouraged to reflect on their actions and experiences to aid in the learning process [1].

There are many advantages to using peer evaluation in a collaborative learning environment. For example, the literature reports that the use of peer feedback can foster individual responsibility and motivate students to provide high-quality work [6, 7]. It has also been shown that peer assessment can increase team members’ accountability to one another and aid in the development of problem-solving skills [5]. A meta-analysis showed several positive results on the effectiveness of peer assessment on quality of learning, such as increased confidence in one’s performance [8]. Peer feedback also offers students the opportunity to reflect on their own work and to compare their work with that of others, which enhances students’ meta-cognitive perceptions [9].

While there are many advantages of using peer feedback in TBL, it must be done well in order to obtain positive outcomes. Students often do not know how to provide effective and appropriate feedback to their peers, as it is not a common experience throughout their educational journey. Inappropriate feedback is not conducive to learning, and may even lead to an environment of mistrust and competitiveness [10]. Students need to learn that feedback should be informative, non-condemning, or non-judgmental; otherwise, it may lead to embarrassment or anxiety, and potentially interfere with relationships among fellow learners [9]. Learning to provide quality feedback is particularly important in medical education, because as medical trainees progress through their training, they will have supervisory responsibilities over more junior learners and they will be expected to provide constructive feedback to these junior colleagues.

Despite this abundance of evidence supporting the benefits of peer feedback in the team setting, there is significant variation among learners’ acceptance of peer evaluation. Some studies have shown that students believe they benefited from peer evaluation, whereas others suggested that students were more resistant to the peer evaluation process [5]. To our knowledge, there is limited information on medical students’ perceptions of the peer feedback provided in TBL, both in terms of its value and how it has affected them as they move forward in their careers as medical professionals. In addition, most studies that examined medical students’ perceptions of peer feedback have utilized questionnaires which have not provided an in-depth look at what students have gained from the experience. A better understanding of those perceptions, and how they might evolve as trainees progress in their education, could identify opportunities for improvement on how students learn to receive and provide feedback.

Oakland University William Beaumont School of Medicine (OUWB) utilizes a well-developed TBL peer evaluation system. In an effort to support student success and enhance team performance, students are asked to provide feedback on peers’ cooperative learning skills multiple times throughout their preclinical years. To help ensure that high-quality feedback is provided, OUWB faculty volunteer to provide qualitative reviews of the students’ TBL peer feedback. The primary goals of this study were to evaluate students’ perceptions of their TBL peer feedback experiences throughout medical school and into residency, and to identify elements of the instructional processes on peer feedback that might be particularly challenging for trainees. This information could help guide the implementation of similar training programs at other institutions. The project was informed by the phenomenology of practice where researchers seek to clarify the meaning that the lived experience of a phenomenon has for participants [11]. Using focus groups and thematic analysis, we aimed to identify how students felt about the TBL peer feedback process, and to what extent they believed that learning how to provide quality peer feedback during the first 2 years of medical school assisted them in providing valuable feedback during their clerkships and residency training. This qualitative approach provided us with an opportunity to thoroughly examine students’ perceptions about the effectiveness of providing and receiving peer feedback during and after medical school.

## Materials and Methods

The theoretical framework of this research was based on social constructivist theory formulated on the original work of Vygotsky (1978) [12]. In TBL, reflection is required throughout many steps of the process. Students use reflection to compare their understanding of the material to that of their teammates’ while solving problems together, as well as when providing and receiving peer feedback [1]. Students connect their prior experiences and knowledge to new material to support their learning [13].

### Peer Feedback Process

During Orientation Week at OUWB, the first-year medical students participate in a TBL designed to introduce them to the TBL pedagogy, as well as teach them how to provide appropriate and valuable feedback to their peers. After experiencing numerous TBLs in the first semester, students are required to provide feedback to their teammates by responding to both Likert scale and narrative questions. For each of their teammates, they are expected to state the single most valuable contribution of their team member and the most important thing their teammate could do to improve the team’s performance. While the students receive feedback from their teammates, they also receive faculty evaluations of the quality of the feedback that the students provide to each other. Faculty volunteers judge the quality of every student’s feedback, scoring it by responding to Likert scale responses and providing a narrative assessment. The written, qualitative feedback provided by faculty is expected to follow the same guidelines provided to the students. This process is repeated at the end of each term during the first 2 years of the undergraduate program, with the only difference being that students only receive Likert scale scores.

### Data Collection

The Oakland University Institutional Review Board determined this study to be exempt from review.

This study utilized focus groups to allow for deeper understanding of students’ perceptions about the effectiveness of giving and receiving peer feedback, rather than a superficial description from a larger sample with a survey design. The focus group facilitator was provided with a guide that included all necessary information to conduct semi-structured interviews, including examples of questions to ask and topics to explore during the session. Those topics included the influence of peer feedback on team performance, student preparation in providing quality feedback, the effectiveness of the peer feedback process, and the value of feedback provided by faculty. Participants were asked to share their overall experience throughout their time at OUWB, with their comments sometimes raising new topics as is often the case with the use of semi-structured interviews. Since the focus of this qualitative study was on the lived experiences of participants, all topics introduced by students and alumni were considered valid.

Inclusion criteria for participants were being medical students in their first through fourth years of medical school at OUWB, as well as medical residents who have graduated from the same program. There were no exclusion criteria. All participants were recruited and informed of the study via email. The investigators arranged focus groups with a maximum of 12 participants in each. With the exception of medical residents, each focus group included only students from the same graduating class for scheduling convenience. For example, one focus group included only first-year students and another was exclusively second-year students. All undergraduate and graduate medical students participated in the Peer Feedback TBL session during their Orientation Week to the undergraduate medical program. Each student provided feedback to their teammates at regular intervals during their M1 and M2 years, which was then evaluated by faculty.

Focus groups were held in private rooms. A standardized open-ended interview approach was used. Although the questions were predetermined, the participants were given the opportunity to answer the questions as they wished. The facilitator was given permission to deviate from the script to ask for clarification to minimize the extent to which the researchers would have to interpret meaning from the transcript. For example, the facilitator was able to ask the participants to define terms or to explain what the students’ experiences meant to them. They were also given permission to ask follow-up questions based on student responses when they felt it was necessary, such as asking the participants “why” they were thinking something. The questions were considered flexible. The facilitator was given permission to deviate from the script if the participants introduced new topics related to the research that were not covered in the list of questions provided. In most cases, the majority of the questions were asked. All questions were asked of the first-year (M1) and rising fourth-year (M4) medical students, and all but one question was asked in both of the focus groups with OUWB graduates. For the second-year (M2) and rising third-year (M3) medical students, three to four of the ten questions were asked. All topics introduced were considered during the thematic analysis.

Immediately following the interviews, $10.00 gift cards were given to all participants. Participation or non-participation did not impact their future training at OUWB or our affiliated hospital system. They were free to withdraw from the study at any time without any negative consequences. The focus group proceedings were recorded and the audio recordings were sent to a professional transcriptions service organization. To ensure anonymity, quotes and other qualitative data obtained during the interviews did not include identifying information and names were not recorded.

### Data Analysis

Thematic analysis was performed by all investigators using the step-by-step guide published by Braun and Clarke in 2006 [14]. Two investigators identified themes from each focus group. Disagreements were resolved by the full consensus of the five investigators. Focus groups for each cohort were organized until saturation of themes was reached.

## Results

A total of 48 participants took part in the focus groups. There were 11 first-year (M1), 12 second-year (M2), 12 rising third-year (M3), and 10 rising fourth-year medical students (M4) and three OUWB graduates.

Overall, the students felt that the idea of providing and receiving effective peer feedback throughout the medical school curriculum was a valuable experience. This was indicated by students starting their medical school program, as well as by resident physicians who had recently graduated.

I think that peer feedback is real (sic) valuable and I really enjoy getting it. It’s sometimes hard to give it because you do have to evaluate the other person from a negative light, but if you’re able to find some ounce of truth that you can then share with them that might help them improve, I think that it’s a worthwhile process. (M1)

I think you have to start the process now of practicing giving the peer feedback… A year from right now we’ll be interns, and we’ll be the ones who are expected to give that feedback to the medical students. So, it is good to start as early as you can. Because that’s how you improve, is that by having someone let you know where you did good, where you did bad. So, I think that is the positive. It’s good, even if the implementation needs a little help, it’s good to practice it starting from your first day of medical school, and just getting better and better at it. So that when you’re entering in as an intern, you can provide that feedback in the direction to interns that you’re working with. (M4)

I think it’s a good thing for medical students to start the process of giving feedback from their peers because they’re going to have to do it when they’re residents…. So, I think it’s a nice first process of... I think it’s a good first step in terms of being able to give feedback to someone. I think it’s a nice first step and in a process of lifelong feedback that you’re going to have to give to someone in the field of medicine. (Graduate)

**Fig. 1:**
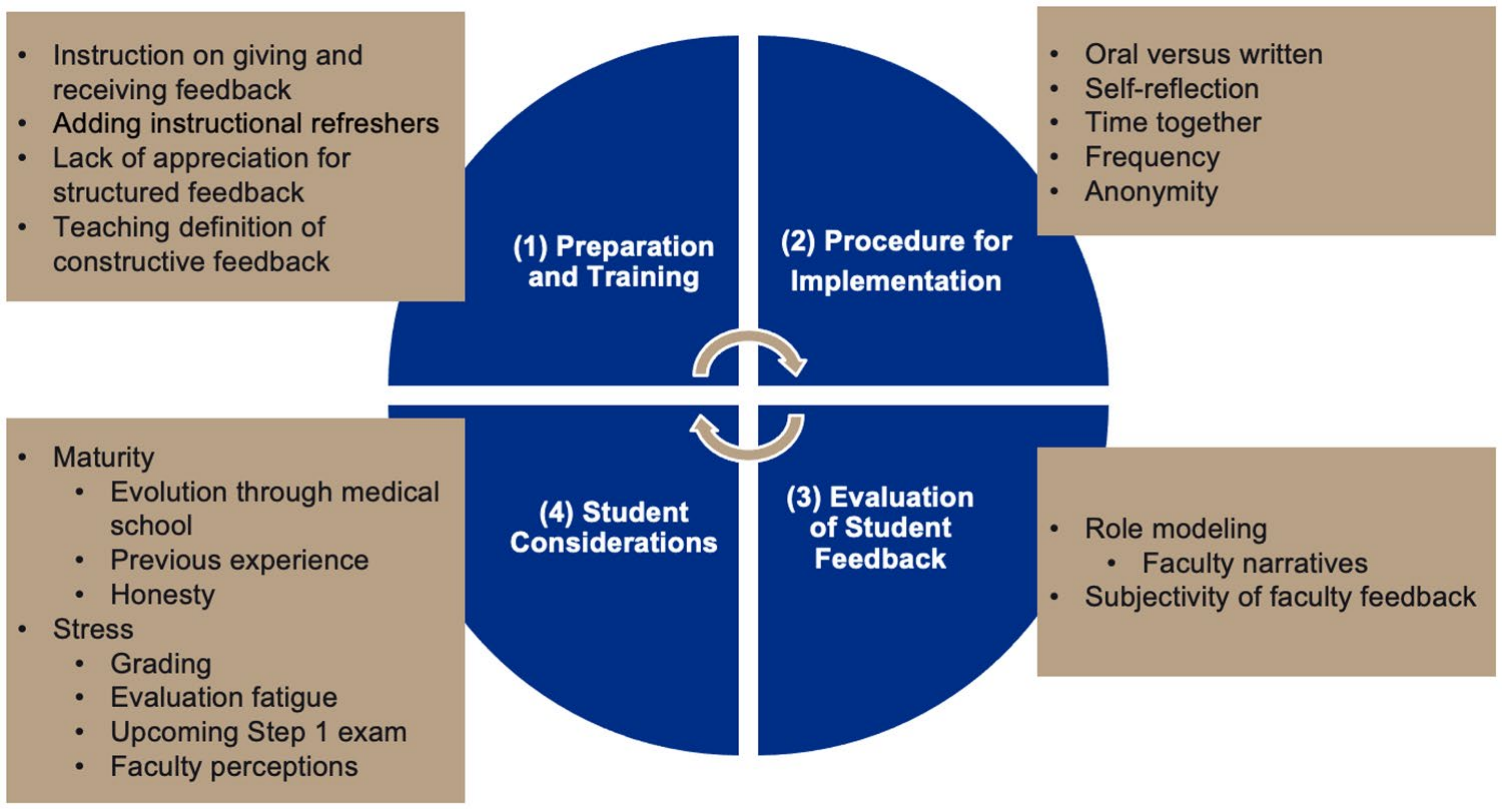
Key themes and interconnected subthemes associated with peer feedback in TBL

There were four key themes and several interconnected subthemes identified during the qualitative evaluation (Fig. 1). The first key theme was preparation and training. A significant subtheme related to this topic was the importance of instruction on how to provide peer feedback as well as how to receive feedback. Overall, students felt it was important to have an instructional strategy on best practices to provide peer feedback. While they felt there were many improvements that could be made to the TBL peer feedback process, they felt the instructional session at the beginning of the medical school curriculum was beneficial. Additionally, they felt a refresher session at the beginning of the second year would also be helpful.

I think we all agree that one beginning session on feedback at the beginning of M1 year was like really important and helpful. Maybe perhaps if we could have another refresher session at the beginning of M2, or maybe like in APM5 right before we get into the clinic, that would be a good thing to have. (M4)

I think the school does do a good job at the start, in only one year, in teaching us how to give constructive feedback and giving us a structure to use, teaching us to use I statements only in our feedback, not saying, “You are,” whatever, and not extrapolating and giving succinct to the point feedback, giving examples. I thought that was good. (M4)

While students felt the instructional session on how to provide effective feedback was beneficial, they did not necessarily appreciate the structure that was required when providing feedback.

And it kind of also becomes just like a cookie cutter where I have this formula now after doing four times, where I’m like, the first sentence is going to be this and my last sentence is going to be, “Great job,” insert the person’s name. One sentence is a specific example, one sentence is, “But you still did a great job.” (M3)

I feel like feedback is important. It’s just the way we’re supposed to structure it, and that kind of makes it difficult to be honest. (M3)

I also think I just wish that there wasn’t a format that I felt like I needed to follow when I was doing it. Obviously, we should be respectful, but other than that, I would like to just be able to be honest. (M2)

In addition, the participants felt that the instructional session should also teach students how to appropriately receive and effectively use feedback.

It’s, one, for self-learning, to get feedback from other people. And then two, being able to provide constructive feedback so you can also keep an eye on or be mindful of things that you can change as well. When you get onto residency and you work out in the real world, you have to be able to work well with others and be able to take and receive and give constructive feedback. So, it’s important to develop those skills early on, whether that’s in a learning kind of classroom setting or whether that’s in a clinical setting as well. (Graduate)

Well also in like the next two years we will get direct feedback from physicians to our faces, and if you can’t handle bad feedback at one point, that’s not going to look very well for you in that clerkship. So it’s something I think that’s important to learn how to handle. (M2)

During the analysis, it was also clear that the definition of constructive feedback needed to be presented more clearly, as many students misunderstood the concept.

I think peer feedback in concept was a really good idea, but I think for what I got, what I heard a lot about it is like, the good comments were generic, the bad comments were made up. Because no one wants to hurt each other’s feelings. Constructive feedback is good, but I think in the TBL setting no one ever wants to hurt the other person’s feelings, so everyone just gives you like, and they’ll tell you like, “Oh I’m just making this up about you.” (M4)

The second key theme was the procedure and implementation of the peer feedback process. One of the noteworthy subthemes included providing oral versus written peer feedback.

I can definitely see both sides, 100%. I think the problem though with doing in like an online format is that it doesn’t get to that level what you were talking about, about truly constructive criticism, because I feel like it allows more passive aggressive or things to be misinterpreted. And like you mentioned earlier, where if somebody says something negative that you didn’t think was an issue, or there wasn’t a communication like face-to-face with that person, then that dynamic between your group or your relationship with that person will then be altered. (M1)

Whereas, I feel like in an open communication, maybe it can start with a survey, and then lead into an open communication where it’s like, “Hey, I put this. This is what I wrote and let’s expand upon it, and let’s discuss, without sugarcoating it,” because at that point, the person has already seen the negative criticism, but then it allows an open discussion where you can vocalize both sides and get to the root of the problem, and try and improve from there. (M1)

Developing that skill of being able to confront someone and have a conversation with them rather than hiding behind a computer screen, it totally changes the dynamic. And I think it would allow people to be more open and honest and also people would take it more seriously, because it’s a face-to-face conversation about something, and it would give you the opportunity to address issues that maybe someone is too scared to address otherwise. (M2)

If getting the feedback was in person, face to face, I took it more seriously. But a lot of the written feedback, I would just gloss over and didn’t really take it seriously. Sometimes I wouldn’t even open the Oasis evals to read my feedback, because I knew it wouldn’t be the most accurate. (M4)

In addition, some participants felt that requiring a self-reflection component would also be valuable.

I think if you’re going to require students to give feedback that people could improve upon, you should require that they do it about themselves so that we’re being reflective in that way rather than other ... even if you’re in a situation where you have to make something up, that’s not something that’s going to happen as much, or won’t be as detrimental if you’re making it up about yourself. (M3)

So, I prefer a place where you’re more self-reflective. A culture where you’re talking about what you can do personally better, rather than saying “well hey, this person ... I have to tell you that you can do this better.” (M3)

The importance of timing also became clear during the analysis. It was noted that the first-year students had a more difficult time with the formative feedback at the beginning of the academic year, as they had spent less time together on teams. With that said, students also noted that it was difficult to remember what their teammates did well or could improve upon; therefore, it would also be helpful to have opportunities for feedback immediately after a TBL session.

It’s really hard to have that many examples of things too, because we don’t spend that much time together, like you were saying. We really don’t. So, we’re very limited on material to even work with. So, to tell someone what they should change, and we don’t necessarily really know. It’s not appropriate for us to say. (M1)

Specifically regarding TBL feedback, if the school really requires us to do written feedback, I think it’d be important to have people give feedback right after TBL. (M4)

Additional subthemes related to procedure and implementation included considerations of how often students were required to provide peer feedback as well as potential concerns with the anonymity of the feedback.

Evaluation of student feedback was the third key theme noted in the analysis, in which the main subthemes included faculty role modeling of how to provide effective feedback as well as the subjectivity of faculty feedback. In general, students felt the evaluation system could be unfair due to the subjective nature of the evaluation process, and in some cases, the inconsistency among faculty evaluators.

Yeah, I think the issue I just had with it when we were being evaluated on the TBL feedback was that, it didn’t seem consistent or objective at all. I mean if you read the comments that the evaluators of the evaluations write, some of them were like this is great, and they give you full points. Then some say, “You didn’t provide enough specific examples.” When you did provide a specific example, but I guess they wanted two or three, whereas that seems excessive. (M3)

A significant subtheme for the fourth key theme, student considerations, included evolving student maturity through medical school. The students admitted they were not always honest when providing peer feedback due to the difficulty in identifying areas for improvement and the fear of offending their teammates. In addition, they were concerned that the feedback provided may cause faculty to view certain students unfavorably.

I won’t lie. My group makes a Google document that you can put examples of maybe when you felt like you didn’t do as well, that I can use. Just because sometimes it’s really hard to find an example. (M2)

We talked about the feedback before writing the feedback, “Okay, I’m going to write this and this, and I think you did this really well. But I think ...” So, we talked about it, and I think that was more efficient and more useful than actually writing it up, and it probably took less time too. (M4)

But it’s like a negative reflection, maybe, from the faculty perspective that this person isn’t fully committed to the team, and they’re always bouncing around. So, there’s not enough context, which I think is a problem. (M1)

Similarly, we found that previous experience was important, since medical residents were grateful for their experiences in learning how to provide high-quality feedback. They more clearly understood why learning how to give and receive feedback is an important skill.

You can take the feedback you learned earlier and you can apply it for your next team. And then looking in the future when you become a resident, you’re going to have to work with medical students and your other fellow residents. I think that taking that feedback in terms of working on a team before you can apply it for whoever you’re going to work with in the future. (Graduate)

As I recall, the peer feedback was pretty helpful. I think it was a good practice for us back then to figure out the best terminology and the best way to approach constructive criticism because that’s pretty important in our field. It helps to get feedback. (Graduate)

In addition, the stress of medical school was an important subtheme related to student considerations. For example, we found that the stresses related to grading, upcoming licensing examinations, and anxiety about faculty perceptions were all factors that affected the peer feedback experience.

## Discussion

Overall, there is a lack of in-depth information related to medical students’ perceptions of peer feedback as they move throughout their career, from first-year students to practicing physicians. The current study employed a qualitative methodology to explore students’ perceptions of their peer feedback experiences throughout medical school and into residency and to determine areas of the process that could be improved to develop a more valuable experience. The findings were organized into four key themes and several interconnected subthemes identified during the analysis (Fig. 1).

### Preparation and Training

The first key theme was preparation and training, in which an important subtheme was the importance of instruction on providing effective peer feedback. Most participants felt the instructional session at the beginning of the medical school curriculum was beneficial. Additionally, the students requested training on how to receive peer feedback. They also felt refresher sessions would be helpful. Providing and receiving feedback is not intuitive and students must be given time to not only learn, but also practice, these delicate social skills [15]. While many instructional sessions focus on how to provide effective feedback, students must also learn how to seek, receive, and handle feedback in order to use it effectively [16]. Unfortunately, the information on receiving feedback is more limited; therefore, educators must make an active effort to provide valuable lessons to help students develop their skills and learn when and how to use feedback effectively [17]. Algiraigri proposed ten tips that help students learn the important skills to properly receive feedback, including self-assessment, controlling one’s emotions, and developing a SMART (specific, measurable, achievable, relevant, and time-bound) action plan [16]. Similarly, Jug and colleagues state that effectively receiving feedback requires clarifying feedback through self-reflection and open communication as well as the development of an improvement plan [17]. While students are often given time to provide peer evaluations, and in many cases receive instruction on how to deliver effective feedback, it is clear that more time should be allotted in the curriculum to allow the development of skills related to receiving feedback, including time for self-reflection and to create an action plan for improvement.

During the analysis, it was also clear that the definition of constructive feedback needs to be presented more clearly, as many students misunderstood the concept. Similar to Burgess and colleagues, we found that students were comfortable providing feedback on what their teammates did well, but were more hesitant to provide feedback on areas for improvement [13]. Instructional sessions on providing peer feedback must make it clear that constructive feedback does not mean it must be negative, correcting, or destructive. Instead, when used effectively, it can improve self-awareness and lead to high-quality learning. Students must realize that it is not disrespectful or ill-mannered to provide constructive feedback, but instead is meant to show one’s peers that they are committed to their success [18]. It should also be emphasized that students must strive for continuous improvement and not stop their efforts once they feel they have adequately met the standards [16].

Lastly, students felt the required structure for providing feedback was inconvenient and unnecessary. Interestingly, Gielen and De Wever examined how the degree of structuring a peer feedback template impacts feedback content and quality, in which they investigated the proportional differences of peer feedback content categories between the no structure, basic structure, and elaborate structure conditions. They provided evidence that suggested the use of a structured peer feedback template can increase the potential impact of peer assessment and improve student learning [19].

### Implementation of the Peer Feedback Process

The second key theme was the procedure for implementation of the peer feedback process. One of the noteworthy subthemes included providing oral versus written peer feedback. Allowing students to provide peer feedback through a conversation may be beneficial and could be a valuable addition to the peer feedback process. For example, Schillings and colleagues completed an exploratory study to evaluate students’ beliefs about peer feedback and to investigate both the instructiveness of face-to-face peer dialogue and the conditions for achieving improved understanding. They found that students perceived face-to-face peer dialogue to be instructive to improve their understanding of written peer feedback and also enhanced their engagement with the feedback [20]. Elnicki and colleagues performed a study to determine if there were differences in how medical residents perceived oral, face-to-face feedback versus written feedback. Overall, no differences were observed between the groups, in which the authors noted that educators could spend more time on other aspects of the feedback process, such as the frequency of the feedback rather than how it is delivered [21]. Overall, while adding opportunities for face-to-face feedback may be helpful and could provide more opportunities for frequent and timely feedback, the research in this area is more limited; therefore, more research on best practices is necessary [20].

In addition, the participants felt that requiring a self-reflection component would also be valuable. This may be an important addition to the peer feedback process, as it was previously noted that self-assessment is necessary for effectively receiving feedback as well [16, 17]. The importance of timing also became clear during the analysis. Students noted that it would be helpful to have opportunities for feedback immediately after a TBL session. Minor adjustments to the TBL process, such as allowing additional time for self-reflection and more timely feedback immediately after a session, could help make the process more valuable for students.

### Evaluation of Student Feedback

Evaluation of student feedback was the third key theme in the analysis, in which faculty role modeling was a notable subtheme. At OUWB, faculty volunteers evaluate the quality of the student narratives, with the intent of “role modeling” effective feedback. Faculty perform this evaluation by using Likert-scale ratings and providing high-quality narrative feedback of their own. Although Likert-scale ratings are provided by faculty several times throughout the year, their narrative feedback is only provided once, due to the time and effort involved with evaluating 125 students’ feedback each academic year. Students appreciated the narrative feedback and would like to receive it more often. Although time consuming, it is beneficial for students to receive constructive feedback from faculty to ensure they are providing effective and informative feedback [15]. In an effort to decrease the amount of time required, process changes could be considered, such as asking students to request faculty feedback when desired or allowing the option for faculty to briefly meet with students to provide face-to-face feedback when requested. The students also stated that the faculty feedback was inconsistent and subjective in some cases. To help minimize these issues, faculty members should be trained on providing effective feedback, with refresher sessions provided as necessary.

### Student Considerations

Student considerations included student maturity and evolution through medical school as well as the stresses of grading, pending licensure examinations, and anxiety about faculty perceptions. For faculty, it is important to keep in mind that students have many other obligations and stressors, both at school and home. This may be especially important when establishing deadlines for the assignments. Unfortunately, the students were not always honest when providing peer feedback due to the difficulty in identifying areas for improvement and the fear of offending their teammates. As faculty, we must make sure students realize that giving feedback is for professional development and to improve team performance, not a personal attack on one’s character. In addition, they were concerned that the feedback provided may cause faculty to view certain students unfavorably. This was similar to the findings of Burgess and colleagues, who noted that students shared concerns about the potential impact on their peers’ academic records or that it may be taken out of context and have unintended consequences [13]. Therefore, it is important that the educational environment be perceived as safe and supportive where feedback is not seen as a threat but as an opportunity to improve.

While the evidence is more limited due to the small number of graduates who participated, we also found that medical residents were more grateful for their experiences in learning how to provide high-quality feedback, as they more clearly understood why learning how to give and receive feedback is an important skill. This may indicate an increase in maturity and professionalism as students move through medical school and into residency. While there is currently not enough research in this area to support this finding, it could be an area to explore further in future studies.

### Study Limitations and Next Steps

The main limitations of our study are that it was conducted at a single institution and was dependent on the voluntary participation of students across all levels of training. Qualitative studies focus on exploring the personal experience of subjects within a specific context. We have aimed to address these limitations by using saturation to guide data collection and thematic analysis to better understand the data. We hope that the description of our local training program will promote the replicability of this project, and that the findings of the thematic analysis guide the implementation of such programs within the educational structure of each medical school. In future studies, we hope to implement mixed-methods designs that will allow us to delve deeper into issues of generalizability. Within our own institution, these results have suggested changes to our training program are needed. Some of our findings identified areas where our curriculum needs to be strengthened (e.g., clarifying the concept of constructive feedback, ensuring the honesty of the feedback, enhancing the quality of faculty feedback). New implementations include the addition of planned redundancy throughout the training program, while extending it into the clinical years. During the latter, training on oral delivery of immediate feedback can be implemented. Finally, creating a parallel training program on self-reflection and accepting feedback are being considered.

## Conclusions

Effective feedback is an extremely important component of professional practice in healthcare and can lead to higher quality care and increased patient safety. For these reasons, it is essential for medical students to learn how to effectively provide and receive feedback. Our analysis raised awareness about several potential areas of concern and difficulty for students in regard to the TBL peer feedback process. Overall, it appears there are many opportunities to educate students regarding the use of constructive feedback. Quality improvement initiatives may include adding opportunities for self-reflection, face-to-face dialogue, and more timely feedback immediately after a TBL activity. One final observation is that for peer feedback to succeed, there needs to be an institutional environment that encourages trust between peers and teachers and is also perceived as being a safe space where such feedback is seen as constructive and in the best interests of all parties. While peer feedback has traditionally been delivered within the context of TBL, its usefulness for supporting the performance of teams and the professional development of their members has led to the ongoing trend of implementing it in other types of collaborative learning experiences [22]. We believe our findings can guide such implementation efforts and future research endeavors in these other instructional modalities.

## Data Availability

Data sharing not applicable to this article as no datasets were generated or analyzed during the current study.
